# Environmental risk assessment of biocidal products: identification of relevant components and reliability of a component-based mixture assessment

**DOI:** 10.1186/s12302-017-0130-0

**Published:** 2018-01-18

**Authors:** Anja Coors, Pia Vollmar, Jennifer Heim, Frank Sacher, Anja Kehrer

**Affiliations:** 1 ECT Oekotoxikologie GmbH, Böttgerstraße 2-14, 65439 Flörsheim, Germany; 2 Department Aquatic Ecotoxicology, Goethe University Frankfurt, 60438 Frankfurt/Main, Germany; 3 TZW: DVGW-Technologiezentrum Wasser, Karlsruher Straße 84, 76139 Karlsruhe, Germany; 4 German Environment Agency, Woerlitzer Platz 1, 06844 Dessau-Roßlau, Germany

**Keywords:** Mixtures, Joint toxicity, Biocides, Wood preservatives, Additives

## Abstract

**Background:**

Biocidal products are mixtures of one or more active substances (a.s.) and a broad range of formulation additives. There is regulatory guidance currently under development that will specify how the combined effects of the a.s. and any relevant formulation additives shall be considered in the environmental risk assessment of biocidal products. The default option is a component-based approach (CBA) by which the toxicity of the product is predicted from the toxicity of ‘relevant’ components using concentration addition. Hence, unequivocal and practicable criteria are required for identifying the ‘relevant’ components to ensure protectiveness of the CBA, while avoiding unnecessary workload resulting from including by default components that do not significantly contribute to the product toxicity. The present study evaluated a set of different criteria for identifying ‘relevant’ components using confidential information on the composition of 21 wood preservative products. Theoretical approaches were complemented by experimentally testing the aquatic toxicity of seven selected products.

**Results:**

For three of the seven tested products, the toxicity was underestimated for the most sensitive endpoint (green algae) by more than factor 2 if only the a.s. were considered in the CBA. This illustrated the necessity of including at least some additives along with the a.s. Considering additives that were deemed ‘relevant’ by the tentatively established criteria reduced the underestimation of toxicity for two of the three products. A lack of data for one specific additive was identified as the most likely reason for the remaining toxicity underestimation of the third product. In three other products, toxicity was overestimated by more than factor 2, while prediction and observation fitted well for the seventh product. Considering all additives in the prediction increased only the degree of overestimation.

**Conclusions:**

Supported by theoretical calculations and experimental verifications, the present study developed criteria for the identification of CBA-relevant components in a biocidal product. These criteria are based on existing criteria stated in the regulation for classification, labelling and packaging of substances. The CBA was found sufficiently protective and reliable for the tested products when applying the here recommended criteria. The lack of available aquatic toxicity data for some of the identified relevant components was the main reason for underestimation of product toxicity.

## Background

Biocidal products are mixtures of one or more active substances (a.s.) and various intentionally added substances serving a broad range of functions in the formulated product. The authorization of biocidal products in the European Union (EU) is regulated by the Biocidal Product Regulation (BPR) [12], and further specified in guidance documents prepared by the European Chemicals Agency (ECHA) in consultation with the EU member states. For the environmental risk assessment (ERA) of biocidal products, the BPR requests considering cumulative and synergistic effects. The methodology to fulfil this requirement is described in the transitional guidance document on mixture toxicity assessment for biocidal products for the environment [13], which is currently available from the ECHA website. In the near future, it will be incorporated into the BPR guidance structure, namely Volume IV Part B and C Guidance, which will then replace this transitional guidance document.

The transitional guidance document particularly explains two different generic approaches for the mixture toxicity assessment. One approach is to conduct ecotoxicity tests with the biocidal product or, e.g. with the environmental mixture resulting from the use of the product (such as leachates from treated wood). The second, so-called component-based approach (CBA) is to calculate the ecotoxicity of the product or of the resulting environmental mixture using ecotoxicity data of the individual mixture components. The concept of concentration addition (CA) is recommended as default for this approach by the BPR as well as by the transitional guidance document, because CA is less data demanding and usually results in a more conservative assessment than the alternative concept of independent action (IA), demonstrated e.g. by Junghans et al. [24]. In comparison to the whole mixture testing approach, the CBA has the strong advantage that it allows assessing the biocidal product and environmental mixtures resulting from its usage with little extra effort, assuming that predicted environmental concentrations are calculated anyway for all relevant product components. Ecotoxicological testing, particularly animal testing, is thereby reduced. The CBA is therefore preferred to the whole mixture testing approach wherever possible [12, 13].

For a reliable CBA, it is essential to include all relevant substances in the calculation. According to the draft Guidance on the Biocidal Products Regulation Volume IV Environment—Assessment and Evaluation (Parts B + C) to be published by the end of 2017 and replacing the transitional mixture guidance, relevant by default are the active substance(s) and all substances of concern (SoC). SoC are defined in Article 3 (f) of the BPR as substances, other than the a.s., which are classified as “dangerous” according to Directive 67/548/EEC [7] or as “hazardous” according to Regulation (EC) No 1272/2008 [11] and that are present in the biocidal product in a concentration leading to a product classification as “dangerous” or “hazardous”. In addition, substances which meet the criteria for being a persistent organic pollutant (POP) under Regulation (EC) No 850/2004 [9], or which meet the criteria for being persistent, bioaccumulative and toxic (PBT) or very persistent and very bioaccumulative (vPvB) in accordance with Annex XIII to Regulation (EC) No 1907/2006 should be regarded as SoCs as well according to the BPR. The BPR further mentions “other grounds for concern” to classify a substance as SoC. Based on the draft Volume IV Part B and C Guidance, SoC identified based on “other grounds for concern” will include

- a.s. from other product types (PTs) contained in the product (e.g. in-can preservatives), provided that a draft final Competent Authority Report (CAR) with an agreed risk assessment is available.
- Product components that intend to enhance the effect of the a.s. in the product (i.e. synergists) as well as organic solvents or surfactants (such as e.g. naphtha) that may enhance the bioavailability of the a.s. and thereby influence its toxicity (on a case-by-case basis).
- Product components that fulfil the criteria for inclusion in the candidate list established in accordance with the REACH Regulation Article 57 (f) and 59 (1) [10], as amended, i.e. endocrine disruptors and PBT substances not covered by Article 57 (d, e).
- Product components that meet two of the three PBT criteria in accordance with Annex XIII to Regulation (EC) No 1907/2006 [10].
- Product components for which an environmental quality standard (EQS) has been established under Directive 2000/60/EC (Water Framework Directive, [8]; according to paragraph 67, Annex 41 VI, BPR).

Applying the CBA in the authorization of chemicals is a rather new procedure that has been formally established for biocides by the BPR. While the predictability of mixture toxicity by CA and the rare occurrence of synergistic (i.e. greater than predicted additive) effects is supported by the literature [3, 5, 27], the reliability of this approach in a regulatory context may still be of concern. Underestimation of product toxicity by the CBA could lead to non-protective regulatory decisions and is therefore of concern for authorities, while overestimation would result in overprotective ERAs. This may appear acceptable from a regulatory perspective, but may be of concern for applicants and unnecessarily limit the availability of biocidal products on the market.

In this context, the objective of the present study was to assess the reliability and protectiveness of the CBA by comparing the predicted with the experimentally determined aquatic ecotoxicity of selected biocidal products. Additionally, the results of this study aim to inform the debate on the identification of ‘relevant’ components for the mixture assessment of biocidal products.

## Methods

In a first step, information on the composition of 21 different biocidal products (product type (PT) 08, wood preservatives) was compiled, and the components were classified for their ‘mixture relevance’ based on their aquatic toxicity following different tentatively defined criteria. The second step consisted in an experimental verification of the predicted aquatic toxicity of selected products, separately for the different sets of ‘relevant’ components established in the first step. The quantification of the deviation between experimentally observed and CA-predicted product toxicity for the different sets of “relevant” components aimed to identify the components that are indeed relevant for consideration in the mixture prediction. The protectiveness of the CBA approach was thereby assessed in comparison to the whole mixture testing approach.

### Compilation of data

For 21 biocidal products with complete dossiers available from the regulatory authorization process in the EU, confidential information on product composition and a base set of data were provided by the German Environment Agency. These data were complemented by retrieving information from the CAR for the a.s. prepared in the course of authorization in the EU, from the ECHA database (http://echa.europa.eu) for those substances among the other components in the formulated products (i.e. the additives) that were registered under REACH, from producer’s safety data sheets (SDS) obtained through Internet search, and from the ECOTOX database (http://cfpub.epa.gov/ecotox) provided by the US EPA. In case of multiple data for the same end point or a toxicity estimate given as a concentration range, the lowest value was used. With regard to aquatic toxicity, median effect concentrations (EC_50_) for invertebrates (*Daphnia magna,* 48 h EC_50_ immobility), fish (various species, 96 h LC_50_ mortality), and green algae (various species, 72 h EC_50_ growth rate) were compiled. If data for the preferred end point were not available, other end points were taken as surrogate such as inhibition of yield in algae, effects in other algal species, immobilization of other crustacean species, EC_50_ for longer or shorter exposure times, and no observed effect concentrations (NOEC). In addition, information on ready biodegradability as defined in guidelines OECD 301 and OECD 310 was compiled for the additives. No quality assessment of the compiled data and no literature search to extend these data available within regulatory processes were performed. Data were compiled for the additives in the base formulations, i.e. pigments were not considered.

### Mixture predictions

Based on the CA concept, the relative theoretical contribution of each product component with the respective toxicity data was calculated in terms of toxic units (TU) and its relative contribution to the sum of toxic units (STU) of all considered components in the product as

1 $$\% {\text{STU}}_{i} = \frac{{{\raise0.7ex\hbox{${C_{i} }$} \!\mathord{\left/ {\vphantom {{C_{i} } {{\text{EC}}_{50 i} }}}\right.\kern-0pt} \!\lower0.7ex\hbox{${{\text{EC}}_{50 i} }$}}}}{{\mathop \sum \nolimits {\text{TU}}_{i} }}*100,$$

with *C*_*i*_ being the maximum allowed concentration of component *i* in the product (mg l^−1^) and EC_50 i_ being the median effect concentration of the component *i* (mg l^−1^). This calculation was conducted separately for each trophic level, i.e. survival of *Daphnia* and fish and growth of green algae using the data compiled in the present study.

According to the CA concept, the EC_50,product_ as toxicity estimate relating to the summed concentration of the considered components was predicted for each trophic level as

2 $${\text{EC}}_{{50, {\text{product}}}} = \left[ {\mathop \sum \nolimits \frac{{{\raise0.7ex\hbox{${C_{i} }$} \!\mathord{\left/ {\vphantom {{C_{i} } {\mathop \sum \nolimits C_{i} }}}\right.\kern-0pt} \!\lower0.7ex\hbox{${\mathop \sum \nolimits C_{i} }$}}}}{{{\text{EC}}_{50,i} }}} \right]^{ - 1} .$$

The EC_50,product_ value was calculated separately for the various sets of selected product components (tentatively identified as relevant, see below) by including only these components with their EC_50,i_ and *C*_*i*_ in the summed concentrations.

### Tentative identification of relevant components

The tentative identification of CBA-relevant components was based on existing regulatory approaches to be as much harmonized with regulatory procedures as possible. It was not the aim of the present project to check the existing assessment or classifications of the selected biocidal products.

Three different sets of criteria were established to tentatively identify additives as ‘relevant’ for a CBA of the product. This was done separately for each product, resulting in multiple counts for additives that were present in several products. All known additives with at least one available end point for aquatic toxicity were allocated to one or more of the established categories (‘HAZ’, ‘CLP’, and ‘> 10% STU’; see below) or to none of these categories. All additives for which aquatic toxicity data were unavailable were assigned to the category ‘no data’, and consequently not included in any mixture predictions.

The category ‘HAZ’ related to the labelling of product components on the product SDS, which is regulated under REACH. According to the relevant guidance [14], all product components shall be listed on the SDS of the product that are classified as “hazardous for the environment” on their own according to CLP and that are either (i) present at concentrations ≥ 0.1% and classified as ‘acute cat1’ or ‘chronic cat 1’, but only if the product is classified, or (ii) that are present at concentrations ≥ 1% and classified as “hazardous to the environment”, regardless of their category and regardless of the classification of the product. As it was beyond the scope of the project to conduct this evaluation for each product, the categorization for ‘HAZ’ depended solely on the product’s SDS. Hence, each product component listed in the product SDS with the respective H- or R-sentence as ‘hazardous to the environment’ was categorized in the present study as ‘HAZ’. In addition, the (non)classification of each product as “hazardous to the environment” was recorded.

The category ‘CLP’ used criteria for the identification of ‘relevant’ mixture components based on the definition stated in Annex I, Part 4 of the CLP regulation [11]. According to this regulation, components identified as relevant should be considered in the classification of the mixture (i.e. the product). This does not necessarily imply that they are listed as hazardous substances on the product SDS, as they may not have directly and ultimately triggered the classification of the mixture (see guidance on the application of the CLP criteria, [15]). While CLP classification relies also on chronic toxicity data and bioaccumulation potential, it was beyond the scope of the present project to compile and include such data in the categorization as ‘CLP’. Categorized as ‘CLP’ in the present study were thereby additives that fulfilled one of the following two sets of criteria:

1. The lowest of the compiled aquatic toxicity end points was ≤ 1 mg/l and the concentration in the product (after consideration of the M-factor according to Table 4.1.3 in Annex I, Part 4 of the CLP regulation, [11]) was ≥ 0.1% w/w.
2. The lowest of the compiled aquatic toxicity end points was > 1 mg/l and ≤ 100 mg/l and the concentration in the product was ≥ 1% w/w.

Consideration of biodegradability in the environment is important for the classification of the ‘Chronic’ categories and, hence, also for the identification as ‘relevant for the mixture’ according to CLP: a component that is rapidly degradable is only considered as relevant for the mixture if its lowest aquatic toxicity end point is ≤ 1 mg/l. However, degradability (in the present study: readily biodegradability) was not taken into account in the categorization as ‘CLP’ or ‘not-CLP’, because excluding readily degradable components from the prediction would interfere with the planned comparison of experimentally observed and CA-predicted product toxicity. Yet, readily degradability as fate-related criterion was included in a second step.

Differences between categorization as ‘HAZ’ and ‘CLP’ in the present study may result from various circumstances such as that SDS may have been issued according to the former directive or that data triggering the classification as ‘hazardous’ by the producer were not available within the scope of the present study for ‘CLP’ categorization. One key difference is that the categorization as ‘CLP’ is more conservative than ‘HAZ’, as it does not require the additive to be present at a concentration that leads to classification of the product as hazardous to the environment.

The third tentative category, ‘> 10% STU’, relates to the predicted contribution of the individual additive to the joint toxicity (sum of toxic units, STU) as predicted by CA. In the context of plant protection products, a substance may be deemed as dominating the mixture if it contributes > 90% to the overall toxicity [20]. Hence, in the present study, a substance was tentatively deemed not relevant if it was predicted to contribute less than 10% STU. Assuming two components in a mixture, and one of them dominating the mixture by constituting 90% of the toxicity, this relates to a maximum cumulative ratio (MCR) [33] of 1.11. The MCR approach provides a quantitative estimate to assess if the mixture as a whole is dominated by one substance (MCR = 1) or equally dominated by all *n* components of the mixture (MCR = *n*). Hence, an MCR could enable a yes/no decision on the relevance of a mixture assessment as such, but does not provide criteria to decide which components need to be taken into account in a CBA.

### Experimental verification of mixture toxicity

Seven wood preservative products with water-based formulations were tested in three different bioassays (products # 6, 10, 14, 16, 20, 22, 23). All products were well miscible with water, and therefore no solvents were used to prepare test solutions. The tested products were either provided by the producer or obtained from commercial suppliers via Web-based shops. SDS obtained along with the products were compared with confidential dossier data (if available) to ensure consistency of information.

#### Algal growth inhibition tests

The growth of the freshwater green algae *Raphidocelis subcapitata* (Culture Collection of Algae at the University of Göttingen) was tested based on OECD guideline 201 [31] using sterilized OECD medium with tenfold increased iron content. All tests were conducted in a climate-controlled room at 21–24 °C. Algae received permanent light at a light intensity between 60 and 120 µE m^−2^ s^−1^. Test vessels were placed randomly on a shaker and constantly shaken with 100 ± 5 oscillations min^−1^. All tests were started with an inoculum of 5000 cells ml^−1^ obtained from a preculture in its exponential growth phase. There were six replicate vessels for the control and three replicate vessels for each of the five test item concentrations, prepared as a geometric dilution series with a spacing factor not exceeding 3.2. After 72 h of static exposure, algal cell density was determined by measuring fluorescence (Multiple Plate Reader Tecan ULTRA). The results (relative fluorescence units) were converted into biomass concentration (cells ml^−1^) based on a calibration curve that was generated from a dilution series of the preculture at the day of the test start. The average specific growth rate was calculated as end point according to OECD 201.

#### *Daphnia* acute immobilization tests

Immobilization (as surrogate for mortality) was tested with the freshwater microcrustacean *Daphnia magna* Straus (clone M 10) according to the OECD guideline 202 [30] using Elendt M4 medium [18]. Test conditions were constant temperature between 19.1 and 21.2 °C and a light intensity between 331 and 607 lx at a 16:8 h light:dark cycle. Two tests were kept in the dark because of suspected photo-instability of one a.s. All tests were started with offspring (less than 24-h-old, at least second brood offspring) obtained from stock cultures kept at environmental conditions similar to the tests. There were four replicate test vessels for the control and for each of the five test item concentrations, prepared as geometric dilution series with a spacing factor not exceeding 3.2. At test start, five randomly selected daphnids were added to each replicate vessel and incubated statically for 48 h. Test vessels were not aerated and test animals not fed during the 48 h. The proportion of immobilized in relation to inserted *D. magna* after 48 h exposure was calculated as end point according to OECD 202.

#### Fish embryo tests

Fish embryo toxicity tests were conducted according to guideline OECD 236 [32]. The eggs used for the test were obtained from an in-house culture of zebrafish (*Danio rerio*) maintained at conditions as prescribed by the guideline. Exposure was started within 1 h of fertilization by transferring the eggs to pre-test crystallizing glass dishes containing the respective test solutions. Eggs were checked for fertilization using a microscope and fertilized eggs were transferred to final test dishes. There were four replicates per treatment, control and positive control (3,4-dichloroaniline, 4 mg/l), each with ten embryos. Five test item concentrations were tested in a geometric dilution series with the spacing factor between 2.0 and 2.2. Test dishes were incubated at a constant temperature of 26 ± 1 °C in a climate-controlled incubator with light conditions of 300–700 lx and a light:dark cycle of 12:12 h. The test vessels were not aerated and the medium was not renewed during the test. The test duration was 96 h in three tests (with product 6, 22, and 23), but 48 h for the other products due to changes in legislation regarding animal welfare during the time of the project. According to OECD 236, the following parameters were used as indicator of mortality: coagulation of fertilized eggs, lack of somite formation, lack of detachment of the tail bud from the yolk sac, and lack of heartbeat. An embryo was scored as dead if one of these four criteria was fulfilled. If scored dead at the daily inspection, the embryo was removed to prevent deterioration and impact on the remaining embryos. Mortality as proportion of dead embryos after 48 h in relation to inserted eggs was used as end point according to the guideline.

#### Chemical analytical measurements

Samples for chemical analysis were taken for the lowest, a medium, and the highest test concentration level from freshly prepared test solutions and in most tests also from test solutions at the end of the exposure period and stored in brown glass flasks at ≤ − 18 °C until analysis. The a.s. and selected additives (as far as analytical methods were available or could be established within the scope of the project) were analysed by direct injection of the samples into a liquid chromatographic system with tandem mass spectrometer (HPLC 1260 Infinity from Agilent Technologies coupled via an electrospray interface to an API 5500 tandem mass spectrometer from AB Sciex). Quantification was done against a calibration in the test medium. Limits of quantification were low enough to detect the residual concentrations of the analytes in all test samples under investigation (except for the blanks).

#### Statistics and evaluation

The observed product toxicity was estimated as the median effect concentration (EC_50_), i.e. the estimated concentration causing 50% effect by means of concentration–response modelling for the key response variables growth rate, immobilization and mortality. Concentration–response was based on nominal concentrations of the product using individual replicates in the software R, version 3.1.3 [34], using the most recent version of the package “drc” [35]. A three-parameter log-logistic model was used for growth rate with the lower limit fixed at 0, according to the function LL.3 given as

3 $$f\left( x \right) = \frac{d}{{1 + e^{{\left( {b*\left( {\log \left( x \right) - \log \left( {{\text{EC}}_{50} } \right)} \right)} \right)}} }}.$$

The parameter *b* describes the steepness of the regression curve, the parameter *d* is the upper limit and EC_50_ is directly modelled as a parameter. The model was reduced to two parameters for binary responses (immobilization and mortality) by fixing the upper limit *d* at 1. The confidence intervals (95%) for all EC_50_ values were obtained with the implemented function “ED” of the “drc” package using the delta method and the *t* distribution.

The observed EC_50_ values for the products (mg product l^−1^) were re-calculated to the sum of the considered components (mg considered compounds/l) for defined sets: (i) only the a.s., (ii) the a.s. and all additives categorized as ‘HAZ’ or ‘CLP’, and (iii) the a.s. and all additives for which toxicity data were available for the respective end point. For this re-calculation, the maximum allowed concentrations of each of the substances in the product were assumed for deriving nominal test concentrations. It is important to note that maximum concentrations are established for each component of a product during the authorization process, while the actual concentration of a component in a marketed product may be lower (in case of the a.s., within a regulatory accepted range that ensures sufficient efficacy). Hence, the exact actual concentrations of a.s. and additives in the tested products were not known, and the maximum allowed concentrations were therefore assumed as nominal concentrations. Still, to verify if deviations of the actual from the maximum allowed concentrations hampered CA predictions, the actual test concentrations of a.s. and some additives were determined and considered in the CA prediction in a second step. To this end, the observed EC_50_ values and CA-predicted EC_50_ values were re-calculated based on measured concentrations (see example calculation in Appendix).

As quantitative measure for the agreement between predicted and observed toxicity, the model deviation ratio (MDR) [1] was calculated for each toxicity estimate of the mixture of the considered product components as

4 $${\text{MDR}} = \frac{{{\text{predicted EC}}_{{ 5 0 , {\text{product}}}} }}{{{\text{observed EC}}_{{ 5 0 , {\text{product}}}} }}.$$

An MDR above 1 indicates that the toxicity of the product is underestimated by the CA prediction based on the considered set of components, while an MDR below 1 indicates that it is overestimated.

Statistical hypothesis testing and correlation analysis were conducted in Statistica, version 12.

## Results and discussion

### Composition of the products and their predicted aquatic toxicity

Based on CA prediction, growth inhibition of algae was the most sensitive end point in the majority of products (Table 1) for which complete information on the composition was available. Products 22 and 23 were selected for experimental testing to represent products where other species than algae were expected to be the most sensitive ones. No complete information on composition was available for these two products. The wood preservative products contained ten different a.s. with fungicidal and/or insecticidal activity at one to four a.s per product. There are currently 40 a.s. authorized in the EU for use in wood preservative products [16], with six of them being based on boron and another six being based on copper. Hence, the selected products do not cover all a.s. in PT08 and their possible combinations, but can be deemed sufficiently representative to draw some general conclusions.

**Table 1:** Active substances, formulation type, labelling as hazardous to the environment according to product safety data sheets, and relative contribution of all additives with available data to the combined product toxicity predicted by CA for the most sensitive end point (in brackets) for each of the wood preservative products

Product	Formulation	Active substances	Product labelled as hazardous to the environment (# HAZ)	Relative contribution (%STU) of additives to the predicted combined product toxicity (most sensitive end point)
1	S	Dichlofluanid (F)	No (0)	11.5 (fish)
2	S	IPBC (F)	No (0)	58.5 (algae)
3	W	IPBC (F), tebuconazole (F)	Yes (1)	37.1 (algae)
4	S	Tebuconazole (F)	Yes (3)	99.8 (algae)
5	W	Boric acid and tetraborate (F, I)	No (0)	34.8 (algae)
*6*	W	Boric acid and tetraborate (F, I)	No (0)	91.6 (algae)
7	S	IPBC (F), propiconazole (F)	Yes (0)	81.9 (daphnid)
8	W	IPBC (F), propiconazole (F)	Yes (0)	0.3 (algae)
9	S	IPBC (F), propiconazole (F)	Yes (1)	62.1 (algae)
*10*	W	IPBC (F), propiconazole (F)	Yes (0)	0.6 (algae)
11	W	IPBC (F), propiconazole (F)	Yes (1)	3.8 (algae)
12	W	IPBC (F), propiconazole (F)	No (0)	0.3 (algae)
13	S	IPBC (F), propiconazole (F)	No (0)	81.9 (daphnid)
*14*	W	IPBC (F), tebuconazole (F)	Yes (0)	1.6 (algae)
15	S	IPBC (F), tebuconazole (F)	No (1)	83.5 (daphnid)
*16*	W	IPBC (F), tebuconazole (F), propiconazole (F)	Yes (3)	31.1 (algae)
17	W	IPBC (F), tebuconazole (F), propiconazole (F)	Yes (0)	1.3 (algae)
18	W	IPBC (F), tebuconazole (F), propiconazole (F)	Yes (0)	5.7 (algae)
19	S	IPBC (F), tebuconazole (F), propiconazole (F)	Yes (0)	90.5 (daphnid)
*20*	W	IPBC (F), tebuconazole (F), propiconazole (F)	Yes (0)	1.0 (algae)
21	W	Boric acid (F,I), fenoxycarb (I), propiconazole (F), fenpropimorph (F)	Yes (0)	97.4 (algae)
*22*	W	Cypermethrin (I)	Yes (0)	0.01 (fish)
*23*	W	Permethrin (I)	Yes (0)	< 0.01 (daphnid)

For products 1–21, mixture toxicity predictions are based on all compounds including confidential additives for which data were available, while for products 22 and 23 predictions relate only to the a.s. and the additives identified in the product safety data sheets. Products indicated in italics were selected for the experimental testing

*S* solvent based, *W* water based, *F* fungicide, *I* insecticide, *# HAZ* number of additives labelled as hazardous (or dangerous) to the environment on the product SDS

Seven of the overall 23 products (Table 1) were not labelled as “hazardous to the environment”, and only 1 of them (product 15) contained one additive that was labelled as such in the product SDS. Hence, these seven products did not contain any hazardous or dangerous additives at a concentration that would result in a labelling of the product as ‘hazardous’ or ‘dangerous’.

In the following, the 21 products will be discussed in more detail for which confidential information on additives was available. These 21 products contained between 2 and 34 additives with a median number of 12 additives per product. There was no significant relationship (Spearman rank correlation, all *p* > 0.05) between the number of additives per product and the predicted combined aquatic toxicity or the predicted relative toxicity contribution of the additives with respect to the most sensitive end point (%STU, Table 1). This is due to the fact that many of the additives were present at very low concentrations (< 0.1% w/w) and/or exhibited low aquatic toxicity (inert compounds such as kaolin). Since none of the additives was labelled as synergist, there was no indication to expect more than additive joint toxicity of the product components, which justifies using CA as the prediction concept [13].

There were eight solvent-based formulations among the 21 products with organic solvents amounting to more than 50% of the total mass (products 1, 2, 4, 7, 9, 13, 15, and 19). All products for which algae were not the most sensitive species were solvent based. The 13 water-based formulations contained organic solvents up to a mass proportion of 7.1%. The predicted combined toxicity (STU) for the most sensitive end point did not significantly differ among water- and solvent-based formulations (one-way ANOVA, *p* > 0.05), but the proportion of predicted joint toxicity contributed by additives was significantly greater in the solvent-based products (one-way ANOVA, *p* = 0.004). Hence, solvents were significantly contributing to the proportion of overall product toxicity attributable to their additives, particularly in the case of *Daphnia*.

In the majority of the 21 products, additives were predicted to contribute at least 10% to the combined toxicity. Additives dominated the toxicity (> 50% STU, i.e. greater toxicity proportion than the a.s.) with regard to the most sensitive end point in 9 out of the 21 products. This clearly indicates that additives can contribute significantly to the aquatic toxicity of formulated wood preservatives based on mixture toxicity predictions and the degree of this contribution varies among products.

### Tentatively relevant additives

**Fig. 1:**
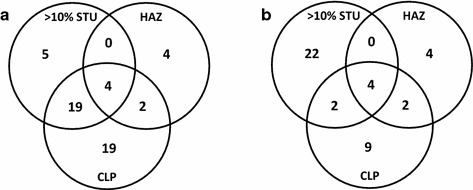
Venn diagram of the three different categories applied to identify additives in the first 21 wood preservative products tentatively as relevant for component-based mixture assessment. **a** Not considering ready biodegradability; **b** considering ready biodegradability for the ‘CLP’ category

The total number of additives in the 21 products amounted to 273. When repeated counting of the same additive in different products was removed, 122 different additives (based on CAS numbers) remained together with 50 ‘unknowns’, i.e. additives with confidential identity or lack of CAS number. For 30 of the 122 different known additives, no data were available for any of the three aquatic toxicity end points. Based on available aquatic toxicity data, about half of the known individual additives (63 of 122) were not assigned to any of the three categories of tentatively relevant additives with regard to their presence in any of the products. The remaining 29 individual additives (53 cases including double counts for presence in different products) were allocated to one or more of the three categories of tentatively relevant components with regard to their presence in at least one product. The Venn diagrams in Fig. 1 illustrate the distribution of the 53 cases. The overlaps between the three categories were rather small with only 25 cases being in any of the four intersections (Fig. 1a). In only four cases, an additive was assigned to all three tentatively defined categories.

The ten additives identified as “hazardous to the environment” in the SDS of the products were all contained at less than 1% w/w in the respective product (Table 2). Six of them were also categorized as ‘CLP’, among them two organic cobalt salts with little contribution to the overall toxicity (< 10% STU). Four of the ten ‘HAZ’ additives were not categorized as ‘CLP’ based on available data. For two of them (a benzotriazole derivative and the organic solvent naphtha), a completed set of aquatic toxicity might eliminate this discrepancy, leaving two cases with a non-overlap of ‘HAZ’ with ‘CLP’.

**Table 2:** Additives in the 21 wood preservative products labelled as hazardous to the environment on the safety data sheet of the respective product

Additive	CAS	Content in product (% w/w)	Categorized as ‘CLP’	Range of % STU (most sensitive species)
3-(2*H*-Benzotriazolyl)-5-(1,1-dimethylethyl)-4-hydroxybenzenepropanoic acid octyl esters	127519-17-9	< 1	No	0.1–0.5
Cobalt 2-ethyl hexanoate	13586-82-8	< 0.5	Yes	0–0.4^b^
Cobalt bis(2-ethyl hexanoate)	136-52-7	< 1	Yes	0–8.0^b^
Cobalt borate neodecanoate	68457-13-6	< 0.5	Yes	0–29.0
Cocodimethylamine	61788-93-0	< 0.1	Yes	n.d.–17.4^a,b^
4,5-Dichloro-2-octylisothiazolinone^d^	64359-81-5	< 0.5	Yes	56.5–88.8
Nonoxynol-10 phosphate	51609-41-7	< 0.5	No	n.d.^a,b,c^
Naphtha	64742-94-5	< 0.5	No	0.1
*N*-(Tallow alkyl)trimethylenediamine, ethoxylated	61790-85-0	< 0.1	No	n.d.–2.3^a^
*N*,*N*’,*N*’-Tris(2-hydroxyethyl)-*N*-tallow-1,3-diaminopropane	90367-27-4	< 0.5	Yes	26.5–53.2

n.d. not determined

^a^No data available for algae; ^b^no data available for daphnia; ^c^no data available for fish; ^d^ authorized as active substance in the meantime

There were five cases (three different additives based on CAS numbers) where an additive was categorized as > 10% STU, but not ‘CLP’ or ‘HAZ’. All these additives contributed less than 20% STU to the overall toxicity, and two of them were readily degradable. Among the 38 cases of ‘CLP’ but not ‘HAZ’ were more additives classified as readily degradable, and 27 of them would be removed from the category ‘CLP’ (Fig. 1b) if rapid degradation in the environment would be applied as additional criterion in line with the CLP regulation [11]. The remaining 17 ‘CLP’ cases comprised mainly organic metal salts and inorganic compounds as well as several preservatives, i.e. a.s. from other product types (all of them with < 10% STU, except one preservative that has become an authorized a.s. in PT08 in the meantime). One of the ‘CLP’ categorized additives, a not readily degradable alkylamine, contributed > 90% STU to all three aquatic toxicity end points and appeared to be erroneously not labelled as ‘hazardous to the environment’ on the product SDS.

Since the ‘CLP’ category showed the greatest number of cases not overlapping with the other categories, it appeared to have the most conservative criteria (Fig. 1a). Yet, applying the criterion ‘ready biodegradability’ reduced the number of cases categorized only as ‘CLP’ to nine, and left the ‘> 10% STU’ criterion as the most conservative category (Fig. 1b).

None of the three tentative categories defined in the present study covers exactly the definition of SoC that should be included in a mixture assessment according to the draft guidance [13] (Biocidal Products Regulation Volume IV Environment—Assessment and Evaluation, Parts B + C). However, none of the additives in these products was suspected of synergistic interaction, identified as candidate PBT or vPvB substances [17], or listed in the Water Framework Directive as priority pollutant with an established EQS. Consequently, the necessity of including such additives in a CBA could not be experimentally verified in the present study. Hence, the only additives to be considered in the 21 products according to the draft guidance definition were a.s. from other PT (some preservatives at very low concentrations), solvents (on a case-by-case basis) and hazardous or dangerous additives that resulted in the product being classified as hazardous or dangerous. In the following, the predicted product toxicity based on the tentative categories will be compared to experimentally observed toxicity, taking also into account the SoC definition of the draft guidance as far as possible.

### Experimental verification of the predicted mixture toxicity

For the experimental verification of the predicted mixture toxicity, five wood preservative products were selected among the 21 products for which confidential information on the additives was available. This selection aimed to include products covering a range of contained number of additives and their categorization as tentatively relevant (e.g. additives expected to significantly contribute to overall toxicity as well as cases where the contribution of additives could not be predicted due to unknown identity or unavailability of data). In the end, however, the selection of products for testing was mainly driven by their availability. Two more products (22 and 23) beyond the 21 products with detailed information were selected for testing to include a.s. with a different mode of action (MoA) and fish or daphnids as the most sensitive species (Table 1). Mixture toxicity predictions for these two products could consequently only take into account the a.s. and the additives identified in the SDS of these products. Only water-based products were selected for experimental testing to reduce any potential interference of organic solvents with the findings regarding compliance with mixture toxicity expectation. Such interference might result from toxicokinetic (synergistic) interactions as pointed out in [4, 36]. Overall, the selected products represented different situations in terms of availability of aquatic toxicity data and the number of additives assigned to the different categories (Table 3). In addition, the products contained a number of solvents (in total less than 10% w/w), preservatives and other additives assigned to none of the three tentatively established categories.

**Table 3:** Wood preservative products selected for experimental testing and number of components (among the known ones) that were identified as potentially relevant for the mixture assessment in the three different categories and their combinations

Product	Active substances	HAZ & CLP & > 10% STU	Only HAZ & CLP	Only CLP & > 10% STU	Only HAZ	Only CLP	Only > 10% STU	None of categories	No data
6	2	–	–	1^a^	–	–	–	1	0
10	2	–	–	–	–	–	2	3	5
14	2	–	–	–	–	1^a^	–	3	5
16	3	2^b^	–	–	1	1^c^	–	7	1
20	3	–	–	1^a^	–	1^a^	–	11	3
22	1	Unknown			–	Unknown		1	Unknown
23	1	Unknown			–	Unknown		2	Unknown

^a^Solvents classified as readily biodegradable; ^b^ additive not classified as readily biodegradable; ^c^ additive classified as readily biodegradable

**Fig. 2:**
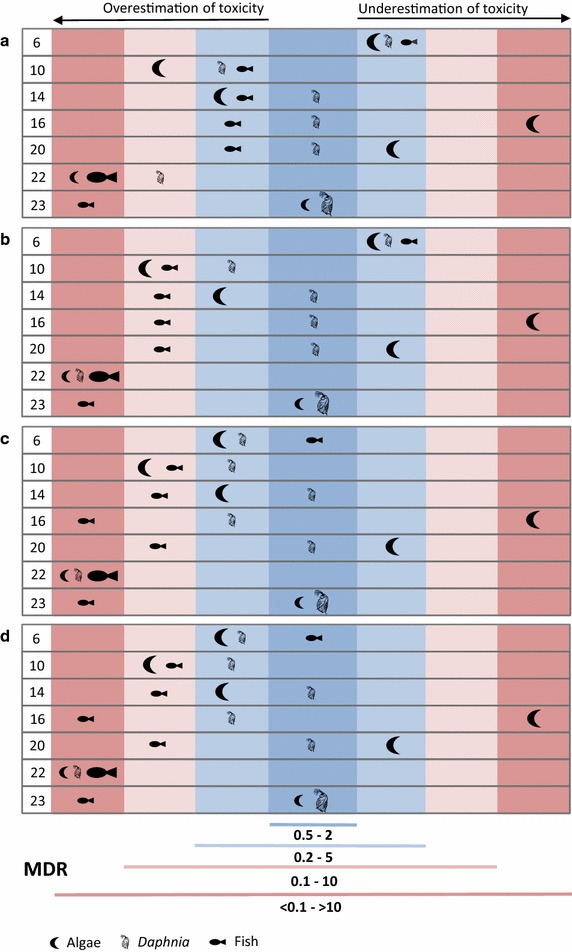
Model deviation ratios for the seven experimentally investigated products based on the predicted and observed toxicity in green algae (ErC_50_), *Daphnia magna* (EC_50_), and fish embryos (LC_50_). Symbols for the most sensitive end point for each product are enlarged. **a** Based only on the a.s. using measured concentrations in the test solutions; **b** based only on the a.s., assuming maximum allowed concentrations in the products; **c** based on the a.s. and all additives categorized as ‘HAZ’ or ‘CLP’, assuming maximum allowed concentrations in the products; **d** based on the a.s. and all additives for which at least one end point of aquatic toxicity was available, assuming maximum allowed concentrations in the products

The experimentally determined deviations from the predictions are shown in Fig. 2 as MDR values determined in the three bioassays for each of the selected seven products. Previous studies generally concluded that a deviation of up to factor 2 is caused by inherent variability and thus acceptable [1–3, 5, 6]. Hence, MDR values in the range of 0.5–2 (dark blue area in Fig. 2) indicate agreement between predicted and experimentally observed product toxicity. Formal statistical comparisons between the predicted and observed mixture toxicity responses have been suggested [22, 23, 37] that could be used instead of a fixed MDR threshold value as a criterion for CA predictability. However, these statistical methods have a high data demand (e.g. observed responses in all replicates for all single-substance and mixture tests), which cannot be fulfilled for the input data available in a regulatory context. Therefore, the derivation of quantitative measures for the degree of deviation (such as the MDR) and an established threshold for this measure as indicating non-additive interaction must be preferred in the context of pragmatic regulatory decision making.

When the CA prediction of product toxicity was solely taking into account the a.s. at their concentrations measured in the biotests (Fig. 2a), the MDR values for the most sensitive end point indicated only for product 23 good agreement with the observed toxicity (MDR between 0.5 and 2). In three other products (10, 22, and 23), toxicity for the most sensitive end point was overestimated by the CA prediction, in one case more than tenfold (fish for product 22). There were three products (6, 16, and 20) with an underestimation of product toxicity for the most sensitive end point (algae). In one of these cases (product 16), the product was more than tenfold more toxic than predicted. CA predictions for other than the most sensitive end points (particularly fish) tended to overestimate product toxicity, except for product 6 where up to fivefold underestimation was also found for fish and *Daphnia*. Considering only the a.s. at their nominal concentrations in the biotests (i.e. assuming the maximum allowed concentrations of the actives in the products) did not change the results for the most sensitive end points, while the trend for overestimation was slightly increased for the other end points (Fig. 2b). This result demonstrated that deviations between nominal and measured concentrations of the a.s. had hardly any impact on the CA predictability.

Figure 2c shows the MDR values when in addition to the a.s. all additives that were categorized as ‘CLP’ or ‘HAZ’ were considered in the predictions. For the MDR values depicted in Fig. 2d, all additives with aquatic toxicity end points were considered for the predictions along with the a.s. Additives were always considered at the maximum concentrations according to product specifications. The concentrations of the additives that were measured in the actual test solutions were either in accordance or below the nominal concentrations (data not shown due to confidentiality of most additives).

The toxicity of product 6 was no longer underestimated for any of the tested organisms when the only additive categorized ‘CLP’ therein was additionally taken into account (Fig. 2c). This demonstrates that this ‘CLP’ additive was indeed responsible for the underestimation when the prediction was based solely on the a.s. Since product 6 was not labelled as “hazardous to the environment” and contained therefore by definition no additives that would lead to such a classification, no consideration of additives in a CBA would be required according to the draft guidance [13]. Since the MDR for the most sensitive end point for product 6 was above 2, but still below 3, and the responsible ‘CLP’-categorized additive was classified as readily degradable, the impact of not considering this additive in a component-based risk assessment would be rather limited in this specific case. However, the example of product 6 clearly illustrates that a CBA using the criteria of the draft guidance can result in an underestimation of product toxicity and thereby possibly lead to unprotective decisions regarding the environment.

The MDR values did not change when more additives than those categorized as ‘HAZ’ or ‘CLP’ (i.e. those categorized only as ‘> 10% STU’ or not assigned to any category based on available data) were additionally taken into account (Fig. 2d). This finding indicates that the consideration of all additives does not improve the predictability of product toxicity, and hence is clearly not necessary. It further shows that the ‘> 10% STU’ criterion alone is too conservative for the identification of additives as relevant for a CBA. Hence, with regard to the draft guidance definition of relevant components, this finding for seven water-based wood preservative products demonstrates that the consideration of a.s. from other product types (here: preservatives) and solvents (unless categorized as ‘HAZ’ or ‘CLP’) was not necessary to predict the overall aquatic toxicity of the product.

Products 16 and 20 represent examples where the consideration of additives categorized as ‘CLP’ or ‘HAZ’ along with the a.s. did not cure the underestimation of product toxicity for the most sensitive end point (Fig. 2c). Consideration of all additives with available data did not improve the predictability either (Fig. 2d). This failure of correctly predicting product toxicity by CA was most likely due to the lack of data for algae (the predicted most sensitive end point) for two of the three additives categorized as ‘HAZ’ (one of them was also categorized as ‘CLP’), while for all of them data were available for *Daphnia* and/or fish, leading to categorizations as ‘HAZ’ or ‘CLP’. The two additives with lacking algal data were both amines (cocodimethylamine and ethoxylated *N*-(tallow alkyl)trimethylenediamine), a group of compounds for which high algal toxicity is known [21, 28] and also reflected in the data set compiled for other amines among the additives. The additives without any aquatic toxicity data in product 16 and product 20 (Table 3) were no amines. Overall, unavailability of aquatic toxicity data for additives regarding the most sensitive end point was the key reason in the selected set of biocidal products that led to underestimation of toxicity in a CBA, even after consideration of additives tentatively identified as relevant.

While underestimation of product toxicity was rare for the selected products, overestimation of toxicity was relatively frequent and increased slightly with the consideration of additives. There are a number of possible reasons for this trend. First of all, nominal (maximum) concentrations were assumed for all product components, while measurements for the a.s. and some additives indicated that actual concentrations in the products were often lower and never exceeded the maximum allowed concentration levels. Secondly, the toxicity data for the single substances can be deemed rather conservative, as (i) exact values were used in case of censored data (e.g. 100 mg/l in case of EC_50_ > 100 mg/l), (ii) the lowest value was used in case of a toxicity range (e.g. 1 mg/l in case of EC_50_ between 1 and 10 mg/l), (iii) NOEC or EC_10_ was used if EC_50_ was not available, and (iv) toxicity estimates for the a.s. were taken from the CAR and thereby represent the most sensitive species for each taxon group, but not necessarily the one used for product testing. Thirdly, concentration addition tends to overestimate toxicity for mixtures of dissimilarly acting components. However, the degree of overestimation by CA compared to IA is relatively small [24] and will likely explain only a small part of toxicity overestimation found in the present study.

Overestimation of product toxicity was particularly evident for fish. This may indicate a generally lower sensitivity of the fish embryo toxicity test (conducted with the products in the present study) compared to an adult fish mortality test (from which the input data for single substances originate). While reported correlations between acute toxicity in fish embryos and adult fish are highly significant [19, 25, 29], the toxicity for an individual substance can easily differ by one to three orders of magnitude between the two life stages [26, 29]. Hence, a deviation between the predicted and observed fish toxicity by about factor 10 appears as within the range of predictability. Life stage-specific toxicity may particularly explain the strong overestimation of fish toxicity for product 22, which contained the pyrethroid permethrin. Pyrethroids are neurotoxic agents, a mode-of-action group that was found to exhibit low or no toxicity towards fish embryos in contrast to the high toxicity observed in adult fish [25]. While results were not conclusive for permethrin in that study [25], the present study does support in line with [26] the lack of correlation between the two fish life stages for the toxicity of permethrin.

Since the present study dealt exclusively with aquatic toxicity, it remains open whether the results and conclusions regarding identification of relevant components and reliability of a CBA could be extended to the risk assessment for the terrestrial environment or human health.

## Conclusions

Overall, the present study provides extensive evidence that a component-based assessment derives sufficiently reliable and, in terms of an ERA, protective estimates for the aquatic toxicity of biocidal products. To achieve this, the consideration of all additives in a product was clearly not required. Yet, the criteria for identifying additives as relevant for a CBA stated in the current draft guidance [13] may need to be re-considered. Including hazardous and dangerous components in the CBA only if their concentrations result in the product being classified as hazardous or dangerous can lead to underestimation of product toxicity (as illustrated by the case of product 6) and thereby possibly result in underprotective decisions. On the other hand, including by default all solvents and a.s. from other product types can lead to overestimation of product toxicity, as illustrated by the comparison with the predictions that included all product components.

Applying the here tentatively established criteria for ‘relevant’ additives (‘CLP’ and ‘HAZ’) would improve the predictability of product toxicity and thereby the protectiveness of the CBA, since additionally those components would be considered that significantly contribute to the product toxicity without triggering the classification of the product as hazardous to the environment. Hence, it is recommended to consider the following components of a biocidal product in a CBA:

1. All active substances in the product.
2. All additives (i) with a relevant aquatic toxicity end point ≤ 1 mg/l and a product concentration of ≥ 0.1% w/w (after consideration of the M-factor according to CLP), (ii) with a relevant aquatic toxicity end point > 1 mg/l and ≤ 100 mg/l and a product concentration ≥ 1% w/w, and (iii) those that are (or should be) labelled as hazardous or dangerous to the environment on the SDS of the product according to REACH.

Based on the here investigated dataset, the components that contribute more than 20% to the overall joint toxicity as well as relevant a.s. from other biocidal product types are covered by these criteria. Using a set of criteria based on the REACH and CLP regulations for the identification of additives relevant for a CBA of a biocidal product has the great advantage to improve consistency and harmonization with the existing regulatory guidance. In terms of workload, the number of additives to be considered would increase compared to the current set of criteria [13]. However, the aquatic toxicity data for the additives need to be compiled anyway in the course of classification and labelling, and therefore no extra effort would be implied for this step of the assessment. In the present data set, the number of additives that would need to be considered in addition to the active substances amounts to 48 in 21 products (i.e. about 2 per product), and is reduced to 21 (about 1 per product, equal to about 8% of all additives) if ready biodegradability is considered additionally.

While a CBA based on such criteria for identifying the relevant components appears to deliver reliable and protective assessments, the greatest deviation between the observed and predicted product toxicity was found to result from unavailability of aquatic toxicity data for some additives (particularly amines) with regard to the presumably most sensitive end points. This underlines the often-stated general constraint of any component-based risk assessment: the result of the assessment critically depends on the availability of data for all relevant components.

## Appendix

See Table 4.

5 $$P_{i} = {\raise0.7ex\hbox{${C_{i} }$} \!\mathord{\left/ {\vphantom {{C_{i} } {\mathop \sum \nolimits C_{i} }}}\right.\kern-0pt} \!\lower0.7ex\hbox{${\mathop \sum \nolimits C_{i} }$}}$$

6 $$C_{{i, {\text{corrected}}}} = {\raise0.7ex\hbox{${{\text{recovery}} (\% )}$} \!\mathord{\left/ {\vphantom {{{\text{recovery}} (\% )} {100}}}\right.\kern-0pt} \!\lower0.7ex\hbox{${100}$}}*C_{{i, {\text{nominal}}}}$$

**Table 4:** Calculation of the predicted EC_50_ for a product considering only the a.s. at their maximum allowed concentrations (nominal concentrations) and calculation of the observed and predicted EC_50_ values considering concentrations of the a.s. measured at three concentration levels in a fish embryo test at test start (initial concentrations)

Parameter	IPBC	Propiconazole	Product
EC_50_ from regulatory source	0.067 mg l^−1^	4.3 mg l^−1^	n.a.
EC_50_ observed in the present study	n.a.	n.a.	96.1 mg product/l
Maximum allowed concentration in the product according to label	3960.4 mg l^−1^	7920.8 mg l^−1^	n.a.
Nominal proportion (*P*_*i*_) in the product, based on maximum allowed concentration	0.3333	0.6667	n.a.
EC_50_ observed in the present study, based on nominal proportions	n.a.	n.a.	1.142 mg sum of IPBC and propiconazole/l
EC_50_ predicted by CA, based on nominal proportions	n.a.	n.a.	0.195 mg sum of IPBC and propiconazole/l
MDR, nominal	n.a.	n.a.	0.17
Recovery (initial measured in percent of nominal concentration), averaged over three concentration levels	91.4%	84.6%	n.a.
Corrected concentration in product, based on initial measured concentrations in test	3620 mg l^−1^	6701 mg l^−1^	n.a.
Corrected *P*_*i*_ in the product, based on corrected concentrations in the product	0.3507	0.6493	n.a.
EC_50_ observed in the present study, based on corrected *P*_*i*_	n.a.	n.a.	0.992 mg sum of IPBC and propiconazole l^−1^
EC_50_ predicted by CA, based on corrected *P*_*i*_	n.a.	n.a.	0.186 mg sum of IPBC and propiconazole l^−1^
MDR, corrected for initial measured concentrations	n.a.	n.a.	0.19

The calculation in each row is based on the information provided in the rows above, using equations 1, 2, 4, 5, and 6

n.a. not available/not applicable

## Abbreviations

ANOVA: analysis of variance
a.s: active substance
BPR: Biocidal Product Regulation
CA: concentration addition
CAR: competent authority report
CAS: chemical abstracts service
CBA: component-based approach
CLP: classification, labelling and packaging of substances
EC: European Commission
EC_50_: median effective concentration
ECHA: European Chemicals Agency
ERA: environmental risk assessment
EU: European Union
EQS: environmental quality standard
HAZ: classification group in present study for additives declared as hazardous for the environment on the product SDS
IA: independent action
LC_50_: median lethal concentration
MCR: maximum cumulative ratio
MDR: model deviation ratio
M-Factor: multiplication factor according to CLP regulation, Annex I Part 4
MoA: mode of action
NOEC: no observed effect concentration
OECD: organization for economic co-operation and development
PBT: persistent, bioaccumulative and toxic
PT: product type
REACH: registration, evaluation, authorization and restriction of chemicals
SDS: safety data sheet
SoC: substance of concern
STU: sum of toxic units
TU: toxic unit
U.S. EPA: United States Environmental Protection Agency
vPvB: very persistent and very bioaccumulative
w: weight
